# Exploring sensitive topics in assessment research through vignette-based interviewing

**DOI:** 10.1186/s12909-026-09891-4

**Published:** 2026-07-08

**Authors:** Sylvia Joshua Western, Jane Hislop, David Hope

**Affiliations:** Clinical Education, Edinburgh Medical School, The University of Edinburgh, Edinburgh, Scotland

**Keywords:** Assessment, Data collection, Medical education, OSCE, Qualitative research, Sensitive topics, Vignettes

## Abstract

**Background:**

Assessment research often involves working with sensitive data, that can mean potential reputation risks to individuals and institutions. Additionally, eliciting emotionally charged personal experiences while protecting the psychological safety of participants can be challenging. While vignettes are increasingly being used in medical education and public health research to elicit data through the presentation of ethical dilemmas, their application to explore sensitive topics, particularly in assessment and more specifically Objective Structured Clinical Examination (OSCE) research, is new. Our research questions were: How can vignette-based interviewing be systematically developed and validated to explore sensitive topics such as test-wiseness in OSCEs? (2) What methodological and ethical value does this data elicitation approach offer for generating insights into this topic?

**Methods:**

Using a five phases model, we have outlined our process of construction, validation, methodological alignment, and deployment of vignettes. We highlight the integration of vignettes with semi-structured interview method and the need for ongoing feedback, reflection and adaptation of vignettes. We undertook thirteen vignette-based interviews with a diverse range of participants, including medical students, graduate doctors, educators with experience in designing, assessing, and running OSCEs, as well as individuals involved in leadership roles and fitness-to-practice panels.

**Results:**

Using qualitative data from our exemplar study, we demonstrate the use of vignette-based interviewing method using four themes: plausibility, dialogicality, flexibility, and narrativity. Outlining example excerpts and analytical insights, we explain how vignette data could strengthen the validity and add insights to interview data, particularly useful in exploring sensitive topics in OSCE research. We have also discussed the advantages of using vignettes within a social constructionist approach, highlighting elicitation of shared beliefs and social norms, and, in terms of interpretation of vignette data, the exploration of competing and complementary constructions and power relations.

**Conclusion:**

Vignette-based interviewing method offers a means to conduct inquiry that is both ethically defensible and allows for a rich investigation, especially valuable in studying topics that are difficult to access through conventional methods. We highlight the importance of careful alignment of selected methods with the overall research design and paradigms, as well as reflexivity and transparency in reporting how and why vignettes were developed and adopted.

**Supplementary Information:**

The online version contains supplementary material available at https://doi.org/10.1186/s12909-026-09891-4.

## Background

Medical school assessments are central to public trust and accountability [1]. Researching assessments often involves studying sensitive topics, such as those which concern academic integrity violations [2]. It is ethically problematic to ask research participants about their behaviours within high-stake assessments which could, for example, potentially lead to disclosure of misconduct. This approach to questioning could have personal, professional and institutional consequences, posing a threat to reputational risk or cause shame to individuals and institutions [3]. Also, studying academic misconduct related topics, for example, exploring certain personality traits that may predict academic misbehaviour [4] or the risk of future disciplinary actions [5], may involve prejudice, moral, and value judgements, necessitating appropriate study design, selection of methods and strategies to mitigate for associated ethical and methodological challenges [2]. Sharing personal experiences of assessments may evoke a strong emotional response, such as stress and anxiety, which necessitates proactive measures to protect participants’ psychological safety [6]. Therefore, health professionals and clinical educators need tools that investigate sensitive topics safely.

Self-reported surveys or questionnaires are commonly used to explore sensitive topics in assessment research, for example, contract cheating [2], plagiarism [7] and academic misconduct [4]. However, these are critiqued for gathering socially desirable responses and under reporting of controversial or harmful behaviours [2]. In qualitative interviewing methods, for example, to study academic misconduct, introducing the topic directly in an interview setting can be confrontational, sensitive, and challenging [3]. Where participants’ recounting of events forms the building blocks of data [8], potential disclosure of certain assessment related information may risk participant safety and confidentiality [9]. This perceived risk may, in turn, inhibit participants’ willingness to share candid accounts [9], thereby compromising the validity of the research method used. In medical education research, vignettes have been used as a data elicitation tool with the purpose of externalising conversations, promoting psychological safety, facilitating the verbal processing of emotions in context, encouraging agency and co-production [10]. While the use of vignettes to explore sensitive topics and ethical dilemmas in medical education research is emerging, their use in OSCE research is particularly novel and timely, especially given the increasing importance of OSCEs in high-stakes medical licensure decisions through the Medical Licensing Assessment (MLA) in the UK [11].

Vignettes are *“evidence- and practice-informed short stories*,* scenarios*,* events or situations in specified circumstances*,* to which individuals or groups are invited to respond*” [12, p.11]. Vignettes, by providing a situationally specific context and a less-threatening third-person space to participants [13], may prevent accidental disclosure of sensitive personal experiences [14]. Vignettes also provide a safe avenue for the ethical use of real-life case studies while protecting the confidentiality of those directly involved in sensitive situations [15]. We have elsewhere discussed eight factors making vignettes especially useful in medical education research [16].

Research across social work [14, 17–19], health and behavioural science [15, 20–22], and higher education [7, 23–25] have used vignette method to explore sensitive topics. In medical education research, for example, a qualitative vignette-based survey has been used to understand how undergraduate medical students dealt with medical errors and their behavioural intentions when confronted with one [26]. Similarly, a vignette survey has been used to explore how medical students’ past encounters of challenging or ethically complex situations influenced their perceptions of acceptable professional behaviours [27]. Further, vignette alongside semi-structured interviewing method has been used to explore how general practitioners and medical students navigated social media use, their behaviours, and their understanding of the threats these pose to their professional and personal lives [28]. In medical education research, vignettes have been used to examine ethically sensitive issues such as professional misconduct and medical errors; however, their use in exploring academic misconduct related topics is limited.

Where vignettes have been used in the wider assessment research primarily through a quantitative approach [7, 29], their use as a means to elicit qualitative data on academic misconduct related topics, is yet to be realised. Previously, a regression analysis, by Geweke et al., used vignettes of hypothetical plagiarism cases integrated into a traditional survey method to explore factors that shaped how students and academic staff at a Swiss university sanctioned and assessed the severity of plagiarism cases [7]. On a similar vein, another study by Rettinger and Kramer, used a survey and experimental vignette method to explore the influence of factors such as cheating culture, neutralising attitudes, and situational contexts that enabled academic dishonesty [29]. While vignette use within quantitative approaches could provide a structured stimulus that could facilitate comparability and allow for cautious generalisations, their application within qualitative interviewing could enable participants to reflect on a focussed context, share their own interpretations and thought processes, and discuss vignette scenarios in an ethically sensitive way [13]. The use of vignette-based interviewing in qualitative research to explore sensitive topics in OSCE research is an area that this study seeks to advance.

The current study forms part of the doctoral work of SJW, which was a qualitative exploration of ‘test-wiseness in OSCEs’, the findings of which are yet to be disseminated. This paper aims to describe the development and validation of vignette-based interviewing method which was utilised within the doctoral research. Using participant responses from one of the vignettes in the doctoral study, we discuss the methodological and ethical affordances of this method as an approach to exploring sensitive topics such as participants’ views on ‘test wiseness’ in OSCEs. Test-wiseness refers to one’s capacity to improve their chances of success in a test by using certain skills or strategies linked to the test format or characteristics, which might not necessarily concern their subject knowledge [30]. Test-wiseness is a sensitive topic as it occupies an ethically complex space between legitimate test-taking strategies and potential academic misconduct, including gaming assessments, exploiting assessment loopholes, and perpetuating inequities in assessments. For example, test-wise behaviours in OSCEs have been described as being *“wishy-washy trying to bluff their way”* [31, p.904] through the exam. Other examples may include the use of external aids for information retrieval in a virtual OSCE and students sharing details of OSCEs through social media, whether inadvertently or intentionally. In the OSCE literature, test-wise behaviours are described as hidden dangers or problematic orientations or unintended consequences of standardisation [32–34]. Given the sensitive, ambiguous, contested, and sometimes adversarial nature of the topic, in which honesty could be challenged, we considered the use of vignette-based interviewing to mitigate some of the ethical and methodological challenges.

## Methods

Prior to commencing the study, ethical approval was granted by the Medical Education Research Ethics Committee (Internal Review Board), College of Medicine and Veterinary Medicine, The University of Edinburgh, Scotland (Ref No: 2022/24). The current study, conducted in 2024–2025, represents a secondary methodological analysis of data collected for a larger doctoral qualitative study exploring medical students’ test-wiseness in OSCEs. The participant pool for the doctoral study included undergraduate and postgraduate students, graduate doctors, educators, staff involved in writing, assessing and running OSCEs, and those in leadership roles and fitness to practice panels at the Edinburgh Medical School. Thirteen participants were recruited using purposive sampling. Participation was voluntary. All participants were informed about the purpose of the study and provided informed consent prior to participation. We used vignette-based interviewing to explore how our participants constructed and made meaning of test-wiseness in the context of summative OSCEs. No additional participants were recruited for the current study. The current study focuses on the participants’ responses to one vignette to investigate the methodological affordances of vignette-based interviewing for exploring a potentially sensitive and socially constructed concept called OSCE test-wiseness.

In this section of the paper, we describe our process of development, validation, integration, piloting and deployment of vignette-based interviewing method. Our construction and use of vignettes for the study was based on the Skilling & Stylianides’s [24] work. While their framework provided a coherent guide to vignette conception, design, and administration within qualitative research methodologies, we describe our approach as a cycle presented in phases to foreground the iterative nature of the process. In extending their framework, we highlight aspects that are less explicitly addressed. In particular, we emphasise the integration of vignettes within interviewing method, and how feedback from subsequent vignette interviews informed the iterative process of vignette construction. We draw attention to continual reflection and adaptation as central to vignette development and use.

**Fig. 1:**
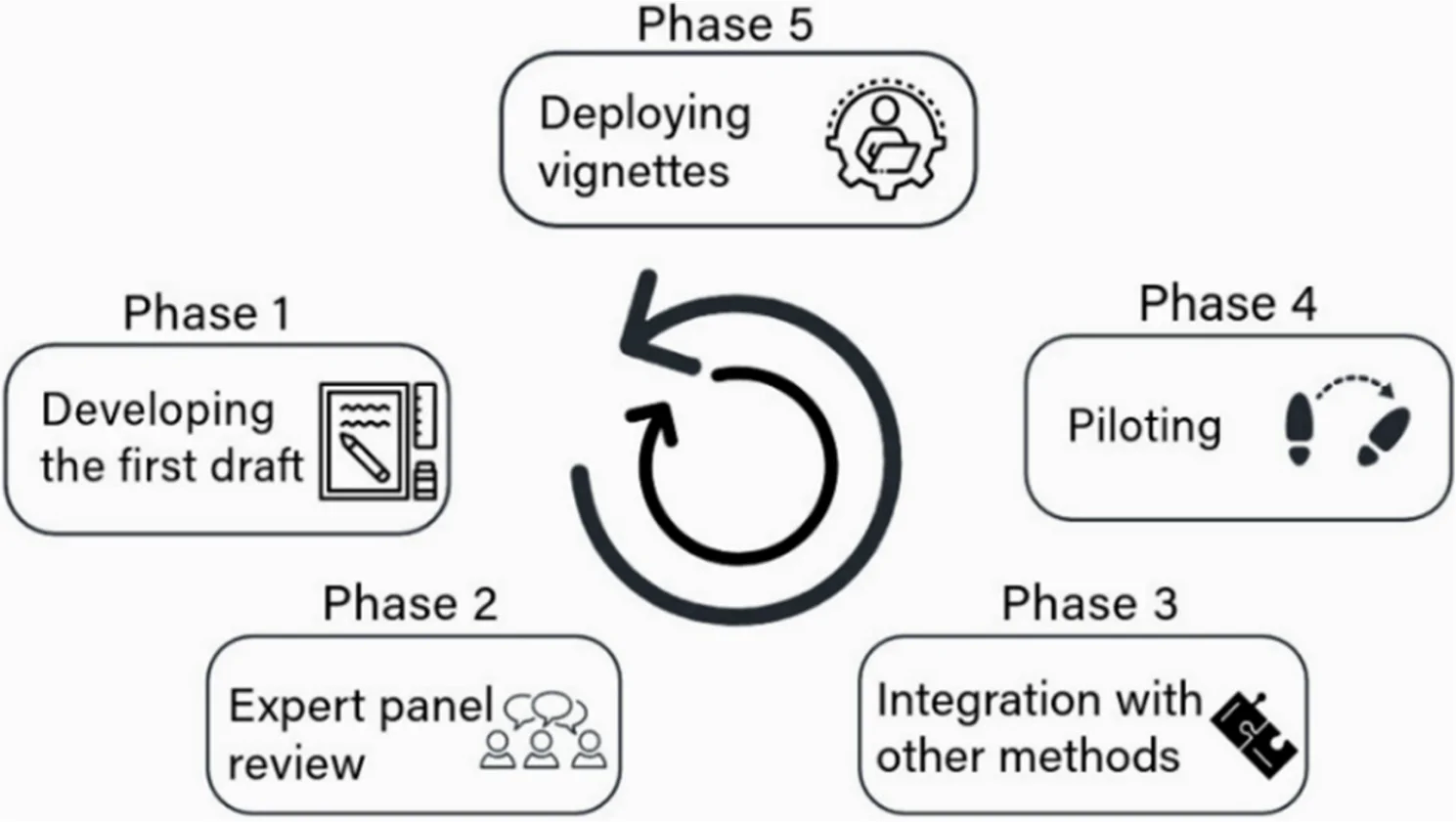
Graphic illustration of the five phases of vignette construction

Beginning with draft zero and progressing toward a refined working draft, the process of developing vignettes was iterative and continuously evolving. Each phase built on the last, allowing for adjustments to be made based on feedback and reflection. Figure 1 depicts a graphic illustration of the five phases of our vignette construction. Phases 1 to 5 of vignette construction outlined in the figure are not meant to be interpreted as strictly cyclical or linear. The clockwise and anticlockwise arrows at the centre of the graphic symbolise the dynamic, back-and-forth movement between phases in the iterative process of vignette construction. This visual representation emphasises the flexibility and fluidity of the process, where continuous reflection and refinement occur as the vignettes evolve.

### Phase 1: developing the first draft

For the purpose of test-wiseness research, we developed five stories inspired by previous personal and professional experiences and shaped them into vignettes (Additional file 1: set of five vignettes). Although derived from real-life experiences, the storyline and characters portrayed in the vignettes were modified such that any resemblance to real-life events or people would be purely coincidental. Table 1 provides a blueprint depicting a broad overview of the five vignettes, the test-wise strategies featured in each, the setting, lead and supporting characters and the topics related to test-wiseness explored. We considered different vignettes to capture a spectrum of test-wise strategies, ranging from ‘desirable’ to ‘undesirable’, which could be set across a range of assessment settings and contexts (formative and summative; online and onsite) and levels of learning (progress exams to licensing), and were appropriate for a diverse range of participants. Topics related to studying the subject content, such as teaching to the test, as well as potential academic misconduct, for example the sharing of OSCE information on social media, were also included. This is because these behaviours could potentially be interpreted as forms of test-wiseness. They were therefore incorporated to capture a range of behaviours, from appropriate to problematic and customised to reflect the nature and boundaries of the concept being studied. The blueprint in Table 1 speaks to our careful development of the different vignette narratives to ensure they were relevant to the concept under study.

**Table 1:** Vignette blueprint

Vignettes	Test-wise strategies	Setting	Characters	Topics explored
Vignette 1: Nat	Kindness as a compensating strategy, Calculated guessing of diagnosis, Presenting a generic treatment plan.	High stakes, summative, Medical Licensing Examinations.	Candidate Nat (international candidate implied), Simulated patient.	Teaching to the test, Gaming the test, Mitigating uncertainty, Influence of exam pressures, Checklist vs. domain marking.
Vignette 2: Jo	Use of external aids for information retrieval, Taking advantage of the virtual format, Strategy for efficient recall.	Virtual online exam, formative context.	Student Jo	Breaking explicit rules, Academic integrity violation, Cognitive offloading, Using assessment loopholes, Pedagogical role of assessments, Role of assessment context (formative vs. summative).
Vignette 3: Gale	Altering performance to suit the assessor’s taste, Conforming to authority, Risk avoidance strategy.	Third-year exam, summative context.	Student Gale, Professor Taylor (Educator and Assessor), Simulated patient (implied).	Ethical conflicts and power dynamics, Influence of hierarchies, Role of assessors.
Vignette 4: Ray and Sam	Sharing or using OSCE information inadvertently or intentionally	Summative OSCE	Student Ray, Student Sam, Friends of Sam.	Academic misconduct, Assessment security breach, Ethical dilemma, Intent vs. Impact consideration, Influence of exam pressure.
Vignette 5: Alex	Using accidentally and unintentionally obtained OSCE information.	Summative OSCE	Student Alex, Student Chris.	Accidental security breach, Intent vs. Impact consideration, role of assessment environment.

The vignette storylines were brief to capture the participant’s attention yet detailed enough to be meaningful. They included abstract details and gaps that stimulated the imagination of participants and provided space to form interpretations and share their understanding and beliefs on familiar topics. Since test-wiseness is understood primarily as the assessment related behaviours of students, the central characters of each of the five vignettes were students or candidates in an exam setting. We chose gender-neutral, common English names for the protagonists. This was to avoid influencing participants’ interpretations of test-wise behaviours through a gender-specific lens. We consciously omitted details on age and ethnicity; one vignette featured an international student sitting a licensing examination to extend our discussion of test-wiseness into cross-cultural contexts.

Methodologically, we used Burr’s social constructionist framework that relocates the focus of meaning making from an individual to the social world of how people interact, the language they use, and the practices they engage in together [35]. We considered the methodological alignment of the vignette storyline, for example we introduced stimulus that could be ethical dilemmas, such as altering one’s medical opinion based on the assessor’s preference in vignette 3 (Table 2). This was intentional so as to elicit shared beliefs and norms, capturing different narratives that might be contradictory or compatible, in line with our social constructionist approach [22]. Writing vignettes involved linking the past, present and the future, thinking back into the past to find stories and thinking ahead into data collection and analysis.

**Table 2:** Vignette 3 (Gale vignette)

Student Gale is a third-year med student performing a summative OSCE today. Through a recent webinar, Gale learnt the current evidence-based approach to treat a health condition was different from what Prof Taylor had taught them in the wards. In today’s OSCE, as Prof Taylor has turned out to be the assessor, Gale says the patient treatment plan in the same way as taught by Prof Taylor, although Gale knows that it is a redundant concept. On a global rating scale, Gale managed to score an ‘excellent’ as Prof Taylor found the performance to be just as it was taught.	

### Phase 2: expert panel review

Each phase was supported by a panel of three assessment experts based at Edinburgh Medical school. The initial drafts of the vignettes were developed by SJW, who also conducted the interviews. Supervisors, DH and JH, who were academics within the medical school, and AJ, who was a senior medical academic with expertise in OSCEs formed the expert panel, helped refine the vignettes by providing feedback on their relatability, clarity of storylines, and whether the behaviours represented a sufficiently diverse range of test-wise behaviours and assessment contexts. The process of vignette construction involved presenting prepared vignettes to the expert panel, seeking feedback and engaging in discussion. This consisted of several iterative rounds of discussion, reflection, consensus-building and adaptation to ensure vignettes were methodologically sound, relatable, plausible and aligned with the research objectives. After drafting, we solicited peer review and feedback on the validity, clarity and relevance of our vignettes from a collegiate external consultant who was experienced in using vignettes in their medical education research.

### Phase 3: integration with other methods

Vignettes have been used within questionnaire surveys [27], alongside one-to-one interviews [36], focus groups [37], or as a stand-alone method [22]. We used vignettes as an adjunct, alongside semi-structured one-to-one interviews. In exploring test-wiseness, interviews on their own was considered inadequate as it was anticipated that participants might feel uncomfortable speaking openly regarding their personal experiences or opinions [3] about their OSCE experiences, while still in the system. Using vignette-based interviewing could create a sense of distance from personal experiences allowing people to share their group or social or institutional norms and also to prompt participants to recall and share similar real-life experiences that they felt were safe to share [13].

To elicit narratives, we used open questions, for instance ‘What do you think about what has happened in this scenario?’ or ‘What do you think of the strategy ___ uses?’. Vignette questions elicited participants’ narratives from varied points of view, such as why they thought a particular test-wise strategy was desirable or questionable or undesirable. These questions focused on the actions and thought processes of the vignette protagonist, exploring the participants’ decision-making pathway, point of view employed, and rationale as they profiled behaviours. The participants’ subjective interpretations of what, why and how the vignette protagonist does or thinks the way that they do, could provide insights into shared meanings and taken for granted assumptions [15]. From construction to implementation, through our engagement with experts and participants in different phases, we recognized that co-construction through social interaction was central to the meaning-making process, aligning with our social constructionist approach.

Integration with the semi-structured interview method involved considerations on the structure and placement of vignettes within our flexible interview guide (Additional file 2). We reflected on the optimal length, the number of events in each vignette, and how they fit within the interview schedule. We deliberated on how many vignettes to select for each participant, as well as how to customise vignette selection to align with each participant’s context and experiences. We opted to introduce vignettes in real-time during the interview to prompt spontaneous reflections of participants’ experiences. While the vignettes were fictional, in terms of distress protocol, participants were informed that they could terminate the interview at any point or choose a different vignette if they did not want to discuss a specific vignette and retract the data within a given timeframe from being analysed [3].

### Phase 4: piloting

Piloting evaluated the plausibility of vignettes. The first pilot specifically focused on assessing the clarity and relevance of the vignette narratives, while the second pilot served as a full trial run, conducted in the same way as the planned main interviews. Both pilot participants had experience in medical education assessment research and were particularly interested in summative assessments. Feedback from pilot testing informed revisions to the vignette, for example, one of the pilot participants found the storyline difficult to follow and often had to stop and revisit sections. For example, in vignette 4 (Additional file 1), revisions included reducing the number of named characters from four to two, deleting a section in which a student explicitly reports the incident to an examiner, and keeping the social media platform generic, simplifying the storyline. Data collected from the pilot interviews were evaluated to determine if the vignettes allowed participants to have meaningful conversations around the concept of test-wiseness. In using the vignettes, we were not seeking to ensure that the participants interpreted the vignettes in the same way as us but would interpret the vignette from their own point of view and construct their own interpretation of test-wiseness. Piloting facilitated development of interviewing skills for the interviewer, including selecting vignettes that best matched participants’ experiences and roles, identifying the optimal timing to introduce the vignettes, when to move away from vignettes to a more generic conversation and vice versa, and asking effective follow-up questions.

### Phase 5: deploying vignettes

Participants were invited to meet with the researcher for a one hour, one-to-one online interview. The vignette-based interviews had an introductory housekeeping and rapport building segment, reiterating the purpose of the research, the interview agenda and informed consent. Participants were asked in general about their OSCE specific roles and relevant experiences. Following which, they were offered two selected vignettes that matched their OSCE roles and experience. Vignette-based questions revolved around the act and thought process of the vignette protagonist, what they would or should do to elicit how people made meaning of and profiled test-wise behaviours.

We introduced vignettes to early participants and sought their feedback. Besides extrinsic feedback on how they found them, the intrinsic feedback from conducting vignette interviews and interpreting the data helped to iteratively refine and adapt vignettes for prospective interviews based on real-time input. This phase explains how insights from one interview could shape the direction of subsequent interviews. The drafting and piloting provided a hands-on experience. Feedback allowed for reflection. Insights from reflections were used to refine and adapt the vignettes. The revised vignettes were tested in subsequent interviews, where the iterative process of refinement and engagement continued.

Social constructionism informed both vignette development and data interpretation. In the current study, using empirical examples of participant responses to a selected vignette, we explored the methodological functioning of the data collection tool, consistent with previous vignette related methodological studies [14, 15, 18, 38, 39]. Generation of four themes relating to the validity and effectiveness of vignette-based interviewing were guided by the goal to illustrate the methodological affordances of vignette-based interviewing, particularly its capacity to elicit insights that may not be elicited through interviewing alone, in an ethically sensitive way. Vignette data was viewed as qualitatively different from real-life experiential data; participant accounts were not assumed as reports of past experiences or predictions of future behaviour, but verbal actions to vignette prompts produced within and shaped by the social context of the interview [15], providing insights into socially situated constructions of reality [22]. The analysis involved data familiarisation and reflexive interpretation by SJW, using vignette literature as a sensitising lens to generate methodological insights. This was followed by critical analysis and iterative discussion among SJW, DH, and JH to refine the themes.

## Results

In this section, we show examples of how vignette-based interviewing helped explore test-wise behaviours of students in the context of OSCEs, presenting a summary of the content only to aid understanding of the examples we give. It is beyond the scope of this paper to discuss our findings on test-wiseness; instead, the focus of this section will be on how vignettes aided in generating discussion of this ethically sensitive topic. Here, we provide selected data excerpts from our interviews specific to vignette 3 (in Table 2) and our interpretations aimed at explaining how vignettes enhanced interview data and why we found the vignette method suitable to explore our topic of interest, test-wiseness in OSCEs. Vignette 3 was purposefully selected as it portrayed a student who prioritised OSCE assessor’s preference over evidence-based practice in an ethically ambiguous situation. Presenting this as a success story prompted reflection on assessment validity and design modifications. The scenario also encouraged discussion of why students might adopt or avoid the behaviour, the varying perspectives of people in different roles, power dynamics, and the hidden influences shaping test-wiseness. This made it well suited to demonstrate the ethical and methodological value of vignette based interviewing. We present our findings under four themes - Plausibility, Dialogicality, Flexibility and Narrativity.

### Plausibility: how far is the vignette relatable?

Postgraduate student Abbie, who is also a medical educator and assessor, responded to vignette 3, saying,

*“I think this is a very plausible scenario*,* particularly in a region where you have a limited pool of tutors and examiners*,* so it’s quite likely that there’ll be some overlap between people who are teaching the students and people who are examining them in their OSCE” – Abbie*

Abbie found the vignette story plausible in a resource restrained setting, highlighting the context specificity of the concept of test-wiseness. For undergraduate medical student Max, on the other hand, although a specific aspect of the vignette (variations in treatment plan guidelines) was not plausible, he could relate to the strategy of acting in a way to please the examiner. Max said,

“*I’m not too sure if that [differences in treatment approach] would happen because I think we’re very much encouraged to focus on national and local guidelines. But I do think*,* when it comes to examination*,* I mean*,* we’re all taught examination by different clinicians. And if you have experience in a clinical environment with a clinician and they’ve said*,* ‘I don’t like it when students do this*,* this is how you should do it’. And you see that clinician as your examiner*,* lot of students would make a conscious effort to please the examiner” - Max*

On one hand, the strategy of adjusting one’s performance to suit the preferences of known assessors was particularly relatable for Max, and that effectively served as a springboard for deeper reflection and discussion on the topic. On the other hand, the point of deviation, in this case, Max, referring to the national and local guidelines that wouldn’t allow for differences in treatment protocol, provided insight into the underlying structures, such as existing guidelines and protocols, that could potentially shape what students do in OSCEs.

### Dialogicality: exploring power relations

After reading vignette 3, senior medical educator Kate mentally stepped into Gale’s shoes, imagining what it would feel like from the student’s perspective, and responded, saying,

*“If I were a student and I had learned something the night before*,* that was really current*,* and I went into the exam*,* and I knew I had been taught by the examiners on the subject a week before*,* I would answer as per what the examiner has taught me. Because I think otherwise*,* the examiners are going to think you’re a noob*,* and you know…exams are playing a game to a certain extent” – Kate*

This excerpt suggests an internal dialogue between Kate as a student and Kate as an examiner. Kate, from a student’s point of view, appears to be concerned about how her responses will be perceived by the assessor, and Kate, from the point of view of an assessor, acknowledges the authority assessors have in what student behaviours are rewarded.

### Flexibility: agency and space for co-creation

By imagining and adding a character ‘Joe’ to vignette 3, Abbie expanded the vignette to illustrate her rationale for thinking of this strategy as harmful, arguing for fairness and equal standards of measurement.

*“If another student to Gale is on the same OSCE circuit and hasn’t been taught by Prof. Taylor*,* say Joe is the other student*,* if Joe has been taught the latest evidence-based approach by another clinician and presents that in the OSCE*,* then*,* is Prof. Taylor going to mark Joe as excellent?” – Abbie*

This shows participants have agency to expand vignettes; now the vignette is a co-created world of the researchers and participants, which aligns with the spirit of social constructionism.

### Narrativity: exploring sensitive complementary and contradictory constructions

Vignettes helped elicit different points of view. Abbie evaluated the strategy from a validity perspective, constructing it as ‘problematic’, thinking through whether that specific test-wise behaviour, if reproduced in a clinical setup, would be harmful or harmless, and for whom. Through the lens of patient safety, Abbie judged it a harmful strategy, saying,

*“Supposing they [Gale] are an FY1 in Prof Taylor’s ward*,* are they going to say*,* ‘Oh well*,* Prof is on call today*,* so we’ll give treatment X’*,* when actually they know treatment Y is better. I think it’s potentially problematic from that point of view.”- Abbie*

Max constructed the same strategy as ‘risky’, which is a complementary construction to Abbie’s, thinking through what if the strategy backfired and did not work as student Gale intended.

“What if the vignette was the other way and the student [Gale] was failed for, sort of, not including something that they would usually do because, perhaps, the assessor [Prof Taylor] followed the marking scheme as opposed to their personal preferences?” – Max

Kate put forth an interesting and contradictory construction with a perspective that it is indeed ‘human nature’ to be performative, viewing it as a common and pragmatic strategy. Kate, said,

*“I probably don’t think it’s harmful. I just think it’s human nature. If I’m a rubbish driver and I never have reversed my car*,* if I can help it*,* but when I go for my driving test*,* I have to please the examiner by reversing the car. Hmm*,* it’s a sort of almost emotional intelligence thing*,* isn’t it?” – Kate*

Vignette-based interviewing allowed us to elicit varied narratives, for example, protagonist Gale’s test-wise strategy in a positive (e.g., ‘emotional intelligence’ or ‘human nature’) as well as a negative view (e.g., ‘potentially problematic’ or ’risky’). We could infer that different people coming from different points of view appeared to construct the same strategy in different ways.

The themes discussed, Plausibility, Dialogicality, Flexibility, and Narrativity, reflected the validity and usefulness of vignette-based interviewing data. They showed how vignettes allowed participants to engage from different points of view and remained open to adaptation and co-construction of the researchers’ vignette narratives.

Importantly, vignettes enabled the elicitation of multiple narratives from a third-person perspective, providing a safe and non-confrontational space for participants to share their perspectives. Participant accounts frequently employed hypothetical and third-person framing, for example, Kate stated, “If I were a student…”, Abbie developed the scenario with “…*if Joe has been taught the latest evidence-based approach*…”, and Max suggested, “What if the vignette was the other way and the student [Gale] was failed for ….”. These examples indicate the use of hedging language and distancing when expressing potentially critical or morally charged views. Vignettes thus elicited hypothetical narratives that allowed participants to frame sensitive issues, such as test-wiseness or strategic exam performance, as vignette-based possibilities rather than personal or professional shortcomings, creating interpretive distance when discussing potentially contentious topics.

## Discussion

This study set out to explore the use of vignette-based interviewing in assessment research involving sensitive topics using exemplar research on test-wiseness in OSCEs. Exploring sensitive topics such as test-wiseness is essential to understand how students and staff involved in assessments make sense of these behaviours, including what is considered acceptable or unacceptable and why, to be able to inform assessment design [40]. Ambiguous boundaries can lead to students constructing their own ethical frameworks in navigating these boundaries, leading to cognitive and emotional labour while also potentially compromising assessment validity [40]. Frequently, qualitative interviews in medical education research require the use of innovative data elicitation tools to explore topics that are difficult to articulate, such as emotionally charged or taken-for-granted routine experiences, where direct questions may be insufficient [10]. This paper highlights the use of vignette-based interviewing as a potential tool in qualitative OSCE research, using storytelling to safely and effectively explore sensitive topics. Further, the paper adds to the methodological discussion on how vignettes function within qualitative interviewing, particularly within the increasingly used social constructionist approach in medical education research.

Previous researchers in health-related disciplines have described vignette construction [18–20, 23, 24, 41, 42]. They have described two major aspects of vignette construction: development and validation. To develop the content, researchers have drawn from real life accounts gathered through: preliminary interviews and studies e.g. [14, 43], personal and professional experience [20, 41], the literature [37, 44, 45], focus group interviews [36], and field or case study notes [39, 46]. The validation of vignettes, they have described as involving consensus building through expert consultation or peer reviews [37, 45], in certain cases using focus groups [36] or interviews [46], using quantitative indices [41] and pre-testing or piloting with people who had similar characteristics of prospective participants [37, 39, 44, 46]. The transparent reporting of the vignette construction process from the inception of vignette storylines to the analysis and interpretation of vignette data is seen as a validation measure, demonstrating a systematic approach to constructing and using vignettes for research [12]. Studies highlight the iterative nature of vignette writing, review, reflection, feedback and adaptation in constructing quality vignettes [36, 41, 44]. Ranging from two to eight steps, several authors have outlined various frameworks, steps, phases or guidelines for vignette construction [12]. We have explained vignette construction as having five overlapping phases loosely based on the framework of Skilling & Stylianides [24]. Development (phase 1), validation with expert consultation (Phase 2) and piloting (Phase 4) form major steps of vignette construction and are discussed widely in the literature. We highlight two other steps that were less explicitly highlighted in their framework - integration with other methods (Phase 3) and deploying vignettes (Phase 5).

Firstly, in adding phase 3 (Integration with other methods), we have created a space for researchers interested in using vignettes to plan ahead and consider how vignettes can function within their methodological framework and align with their theoretical and philosophical assumptions. In the context of the present study, vignettes enhanced the data obtained by tapping into areas not explored through the interviewing method [47]. At certain instances, vignettes prompted participants to discuss similar events from their memory; at other times, participants seemed to interpret the vignette protagonist’s thoughts, words and actions through their own experience in similar contexts. Vignette-based interviewing helped elicit participants’ verbal actions and influences in a given situational context, judgments and rationalisations in the face of moral/ethical dilemmas, and elicitation of sensitive personal experiences while protecting the psychological safety of participants [47].

Secondly, by adding phase 5 (Deploying vignettes), we frame vignette construction as an evolving, ongoing process that spans throughout the data collection process of the research. In previous models, vignette construction is usually complete at the stage of piloting [37]. In the spirit of co-creation with participants, we have proposed a fifth phase, wherein feedback from deploying vignettes in earlier interviews informed the use of vignettes in subsequent interviews. Therefore, vignette construction in a way continued throughout the data elicitation process.

In the results section, by using example excerpts from test-wiseness research, we discussed common methodological considerations, such as plausibility of vignettes; the advantages of using vignette methodology, such as safety and flexibility; and, in terms of interpretation of vignette data, dialogicality and the exploration of narrative constructions of competing and complementary constructions and power relations.

Qualitative literature on vignettes across all fields highlights the importance of plausibility as it is closely related to the validity of the tool; it asks if the story depicted in the vignette is representative of the phenomenon being explored [48]. Researchers play a key role not only in the process of designing plausible vignettes but also in evaluating how plausible the vignettes are to the participants [19]. For a vignette to yield rich data, it should be designed in such a way that it invites participants to imagine themselves as vignette characters, putting themselves in the character’s shoes [15]. Participants’ accounts presented in the results section evidence plausibility of vignettes, and hence the validity of the research method employed. However, as described, we acknowledge the impossibility of constructing vignettes where all the developments within the scenarios are viewed as highly plausible by all participants [15]. It is useful to think of deviations of participants’ responses (from pre-determined responses of the researchers) as desirable aspects rather than threats [15]. As explained before, participant Max’s response regarding the implausibility of a particular aspect of vignette 3 provided an opportunity to gain insights into the existing structures that prevented the story from developing in a predetermined direction. Therefore, determining whether a participant finds a vignette plausible goes beyond a simple yes/no answer; it appears that there are various ways in which participants could relate to the specifics of the vignettes, and gaining an understanding of that is useful.

In understanding how participants engage and interpret vignette stories, it might be useful to consider plurality of voices within vignette data [49]. We can explore the nature of dialogue between the differing positions of the self and the other in interpreting vignette data using the theory of dialogicality [49]. The theory, instead of viewing individuals as possessing a singular, fixed identity, allows us to see the vignette data as embodying multiple voices that reflect various social and personal identities [49]. As shown in the results section, the vignette data helped explore the different roles participant Kate played and the dialogue between these positions that may have shaped her understanding of test wiseness. Exploring dialogues within vignette data helps us to understand power relations in context as well, asking what and who constrains or enables behaviours.

Vignettes, unlike real-life events, allow ambiguity that allows flexibility, adaptation and expansion of stories [47]. Our participant’s prediction of how the vignette story would unfold could reveal taken-for-granted assumptions that are the aggregated product of the participants’ social experiences [15]. This speaks to the agency and co-construction of narratives that vignette based interviewing promoted.

Using a social constructionist framework, we can explain how vignettes help elicit valuable insights into narratives that can either support or contradict each other [22]. These narratives may refer to reality in unique ways and are shaped by the social and cultural contexts in which they exist [22]. While other methods, such as interviews, can very well help elicit accounts of such contradictory and complementary narratives, what vignettes offer is a context that is a realistic scenario and a non-confrontational, safe space for participants to relate to people and events in the vignette without the fear of losing face. In researching test-wiseness, a concept that is ambiguous and difficult to define, vignettes offered concrete situational contexts that seemed helpful as entry points to discuss behaviours and rationales safely in an ethically sensitive way.

From a methodological point of view, social desirability is a commonly cited concern even with vignette usage, that is, the participant responses may not accurately reflect their real self but rather a socially acceptable version of themselves [14]. Unlike pure realist researchers, who may be concerned that the data may not reflect the real self, social constructionist researchers are rather interested in the socio-cultural meanings and discourses people draw on [50] when responding to vignettes. Considering the inseparable nature of individual views and their socially sanctioned versions, vignette responses can be seen as a mix of self-disclosure, socially stigmatised views and repeated socially approved explanations [49]. Instead of viewing social desirability as a threat, we approached it differently by writing vignettes that focused on ethical or moral dilemmas in a way where there may not be a clear right or wrong response. Socially desirable responses then could be seen as default and an opportunity to explore the underlying value-based, taken-for-granted assumptions that shaped behaviours.

### Strengths, limitations and practical implications

For researchers using or exploring the use of the vignette method, we provide exemplar which could help inform their own research design and decision-making. In assessment research, this paper opens pathways to explore sensitive topics safely, and consider vignette use within other research designs (e.g., longitudinal studies) or paradigms (e.g., post-positivist or constructivist) or across different modes of vignette delivery (e.g., videos or photographs).

Participant responses may have suffered response bias, a common concern in most qualitative explorations. To encourage a balanced discussion, we had written vignettes in such a way that the ethical or moral dilemmas mentioned may not have a clear right or wrong response. Nevertheless, given the sensitive and morally charged nature of the topic, it is understandable that participants might have been reluctant to discuss experiences that might be perceived as personal or professional shortcomings.

While we offered a retrospective account of vignette construction and use, future research could adopt a prospective approach, for instance, in the UK Medical Licensing Examinations context, stakeholder-constructed hypothetical vignettes on test-wiseness could proactively anticipate challenges, promote co-creation and explore the complex decision-making processes, and propose solutions well ahead of the game.

## Conclusion

In this paper, we bring to light the process of how we constructed and used vignette-based interviewing as a data collection method in our research that explored a sensitive topic in assessment research: test-wiseness in the context of OSCEs. This is a novel contribution to medical education research in two ways: (1) introducing vignette-based interviewing as a potential data elicitation tool for use in assessment research, especially OSCE research (2) detailing the iterative process of construction and use of vignettes within a social constructionist approach. We have presented vignette construction in five phases, emphasising two key phases that were less considered in the literature: Integration with other methods and Deploying vignettes. We have discussed vignette usage under four themes: Plausibility, Dialogicality, Flexibility and Narrativity. These themes explained the different ways in which vignette data can add insights to the interview data, especially in an ethically sensitive way. We advocate for the broader adoption of this tool by assessment researchers, especially those who are interested in investigating ambiguous and ethically sensitive topics.

## Supplementary Information

Additional file 1: Set of five vignettes used.

Additional file 2: A flexible interview guide.

## Data Availability

Due to the sensitive nature of the qualitative data and to protect participant confidentiality, the data is not publicly available but may be available from the corresponding author upon reasonable request and with appropriate ethical approval.
