# Supplementary material for: Exploring sensitive topics in assessment research through vignette-based interviewing
Source: BMC Med Educ. 2026 Jul 8;26:1552. doi: 10.1186/s12909-026-09891-4 (PMC13629074; doi:10.1186/s12909-026-09891-4)
Supplement: Supplementary file 1 [file 12909_2026_9891_MOESM1_ESM.pdf]

Additional file 1: Set of five vignettes used

Vignettes	Content of the vignette
Vignette 1:<br>Nat vignette	Nat is appearing for a medical licensing examination in the UK. Nat has learnt and practiced several OSCE scenarios and has taken a couple of mock OSCEs from a renowned coaching center. One of the tips Nat learnt was to make sure to listen and talk nicely to the patient in a kind manner.<br>On the day of the exam, Nat is tasked to provide diagnosis and treatment counseling to a simulated patient. Unfortunately, Nat is unsure of what the patient's problem is. Nat remembers the kindness tip taught in the coaching center and makes sure to nod and smile while listening to the patient. Nat takes a calculated guess on the diagnosis, presents a broad generic treatment plan to the patient in a kind manner, and completes the OSCE task. Although Nat could not check most of the criteria expected out of the checklist, on a global rating scale Nat managed to get a borderline pass in that station.
Vignette 2:<br>Jo vignette	Jo is taking a virtual formative OSCE exam today. The students are required to engage with the formative OSCE just as they would do with a summative one. While studying for the exam, Joe prepared post it notes with salient details on all possible health conditions within the scope of the subject content assessed and attaches them around the edges of the monitor. Jo frequently refers to these notes while performing the OSCEs.
Vignette 3:<br>Gale vignette	Student Gale is a third-year med student performing a summative OSCE today. Through a recent webinar, Gale learnt the current evidence-based approach to treat a health condition was different from what Prof Taylor had taught them in the wards.<br>In today's OSCE, as Prof Taylor has turned out to be the assessor, Gale says the patient treatment plan in the same way as taught by Prof Taylor, although Gale knows that it is a redundant concept. On a global rating scale, Gale managed to score an 'excellent' as Prof Taylor found the performance to be just as it was taught.
Vignette 4:<br>Ray and Sam vignette	A summative OSCE is designed to be conducted in two consecutive circuits for 24 students, with 12 students in each group. After completing the exam, student Ray from Group A posts a message on their social media, "Tired after CPR station #oscedone&dusted".<br>Student Sam from Group B saw this post seconds before they were asked to submit all their electronic gadgets. Student Sam then revises the CPR procedure with two other friends in Group B, who has no idea about this social media post. Group B students are called into the OSCE exam hall. Sam and friends performed well in the CPR station.
Vignette 5:<br>Alex vignette	During a summative OSCE assessment, student Alex is in a rest station and student Chris is in a nearby active station where a patient counselling OSCE is going on. As these are happening in adjacent rooms and the exam zone is otherwise silent, student Alex can hear student Chris to a certain extent. Alex gets to hear some words which Chris uses and is thinking about what OSCE station it could be. The bell rings and Alex goes into the active station.
