# Supplementary material for: Exploring sensitive topics in assessment research through vignette-based interviewing
Source: BMC Med Educ. 2026 Jul 8;26:1552. doi: 10.1186/s12909-026-09891-4 (PMC13629074; doi:10.1186/s12909-026-09891-4)
Supplement: Supplementary file 2 [file 12909_2026_9891_MOESM2_ESM.pdf]

Additional file 2: A flexible interview guide

1. **Introduction and Consent Process**
    - Introduction and housekeeping.
    - Provide an overview of the study and its purpose to the participant.
    - Participant Information Sheet and Consent Form: Ensure participant has returned signed informed consent for participating in the study.
    - Reiterate the participant's right to choose not to answer any questions or to terminate the interview at any point if they feel uncomfortable.
    - Consent for Recording: Obtain explicit consent for audio recording of the interview.
2. **Exploration of participant's background and OSCE Experience**
    - Can you share your experiences with OSCE assessments?
    - Age band, current roles - Your current role(s): undergraduate student, postgraduate student, medical education fellow, medical educator, OSCE assessor, OSCE station designer, involved in fitness to practice decisions, administrators, course coordinators, leadership roles, Number of years of experience with OSCEs (include both student and academic experience), (3-5 years) (6-10 years) (>10 years)
3. **Test-wise behaviours broad**
    - Giving an example of test-wise behaviour - checklist or handwashing, ask What are your thoughts on that?
4. **Possible follow-Up Questions**
    - Could you elaborate on that [their take on test-wise behaviour in the example context]?
    - Describe the most recent occurrence of this [follow up on any example test-wiseness they give].
    - Could you talk through why you think this is a useful and healthy/harmful/harmless behaviour?
    - How did you feel/react/respond in that situation?
    - How did others involved react/respond, and what were the outcomes?
    - How did you learn to manage these challenges, and what influenced your decisions?
    - Are there any explicit or tacit guidelines or practices in your workplace exist that pertain to test-wise behaviours?
5. **Vignettes**

- Vignette selection (pick any two depending on the roles and experience of the participant)
- Facilitate discussion on participant perspectives regarding each vignette, focusing on behaviours, consequences, and desired responses.

Vignette 1:

1) What do you think about what has happened in this scenario?
2) What do you think about Nat's behaviour?
3) If you are Nat's assessor - what are your thoughts on the strategies Nat has used?
4) How do you think this could affect the purpose of this assessment?
5) If this happened in your place of study or work, how would you like it to be addressed?

Vignette 2:

1) What do you think about what has happened in this scenario?
2) What do you think about Jo's behaviour in this scenario and the circumstances around it??
3) If you are Jo's assessor, what are your thoughts on the strategies Jo has used?
4) How do you think this could affect the purpose of this assessment?
5) If this happens in your place of study or work, how would you like it to be addressed?

Vignette 3:

1) What do you think about what has happened in this scenario?
2) What do you think about Gale's behaviour in this scenario and the circumstances around it?
3) If you are assessor, what are your thoughts on the strategies Gale has used?
4) How do you think this could affect the purpose of this assessment?
5) If this happens in your place of study or work, how would you like it to be addressed?

Vignette 4:

1) What do you think about what has happened in this scenario?
2) What do you think about Sam's behavior in this scenario and the circumstances around it?
3) If someone has reported this to you as an examiner, how would you handle this situation?
4) How do you think this could affect the purpose of this assessment?
5) If this happens in your place of study or work, how would you like it to be addressed?

Vignette 5:

1) What do you think about what has happened in this scenario?
2) What do you think about Alex's behaviour in this scenario and the circumstances around it?
3) If you are the examiner, how would you handle this situation?
4) How could this affect the purpose of this assessment?
5) If this happens in your place of study or work, how would you like it to be addressed?

6. **Closing Questions**
    - Is there anything else you would like to share?
    - Do you have any questions for me?
    - Is there anything else you believe is important for me to ask to better understand your experiences with test-wiseness?
7. **Closing Remarks**
    - Thank the participant for their time and insights.
8. **End Recording**
    - Stop the recording and ensure the participant is comfortable with the conclusion of the interview.
